# Clinicopathological Characteristics and Prognostic Factors in Ovarian Metastases from Right- and Left-Sided Colorectal Cancer

**DOI:** 10.3390/curroncol28040255

**Published:** 2021-08-03

**Authors:** Ondřej Kubeček, Jan Laco, Jiří Špaček, Alena Kubečková, Jiří Petera, Iva Selke Krulichová, Aleš Bezrouk, Stanislav Filip, Jindřich Kopecký

**Affiliations:** 1 Department of Oncology and Radiotherapy, Faculty of Medicine and University Hospital in Hradec Králové, Charles University, Sokolská 581, 50005 Hradec Králové, Czech Republic; okubec@gmail.com (O.K.); jiri.petera@fnhk.cz (J.P.); stanislav.filip@fnhk.cz (S.F.); 2 The Fingerland Department of Pathology, Faculty of Medicine and University Hospital in Hradec Králové, Charles University, Sokolská 581, 50005 Hradec Králové, Czech Republic; jan.laco@fnhk.cz; 3 Department of Obstetrics and Gynecology, Faculty of Medicine and University Hospital in Hradec Králové, Charles University, Sokolská 581, 50005 Hradec Králové, Czech Republic; jiri.spacek@fnhk.cz; 4 Department of Gynecology, First Private Surgical Center, Ltd., Sanus Hradec Králové, Labská kotlina I/1220, 50002 Hradec Králové, Czech Republic; gynekolog.hk@gmail.com; 5 Department of Medical Biophysics, Faculty of Medicine in Hradec Králové, Charles University, Šimkova 870, 50003 Hradec Králové, Czech Republic; krulich@lfhk.cuni.cz (I.S.K.); BezroukA@lfhk.cuni.cz (A.B.)

**Keywords:** colorectal cancer, metastasis, ovarian metastases, prognostic factors

## Abstract

Background: Secondary tumors of the ovary (STOs) account for 10–25% of all ovarian malignancies, including metastases from primary gynecological tumors. Colorectal cancer (CRC) has been recognized as one of the most common causes of STOs in Western countries. Despite it being well-known that CRC originating from the right versus left side of the colon/rectum differ substantially, there is a paucity of information regarding the effect of the primary tumor sidedness on the clinicopathological characteristics of STOs. Methods: This retrospective, observational chart review study included patients with histologically confirmed STOs of CRC origin diagnosed between January 2000 and December 2019. The clinicopathological characteristics of STOs originating from left-sided and right-sided CRC were compared. Univariable and multivariable analyses employing elastic net Cox proportional hazard models were used to evaluate potential prognostic factors. Further, the role of imaging methods in STOs diagnostics was evaluated. Results: Fifty-one patients with STOs of colorectal origin were identified. The primary tumor originated in the right and left colon/rectum in 39% and 61% of the cases, respectively. STOs originating from right-sided primary tumors were more frequently bilateral, associated with peritoneal carcinomatosis, had the ovarian surface affected by the tumor, and contained a mucinous component. The independent prognostic factors for overall survival in the whole cohort included: the presence of macroscopic residual disease after cytoreductive surgery, menopausal status, the application of systemic therapy, and the application of targeted therapy. In 54% of cases, the imaging methods failed to determine the laterality of the STOs correctly as compared to pathological reports and/or intraoperative findings. Conclusion: STOs originating from left-sided and right-sided CRC show distinct clinicopathological characteristics. Moreover, different metastatic pathways might be employed according to the primary tumor sidedness. Considering the discrepancies between radiological assessment and histopathological findings regarding the laterality of STOs, bilateral adnexectomy should be advised whenever feasible.

## 1. Introduction

Ovarian metastases account for 10–25% of all ovarian malignancies, including metastases from primary gynecological tumors [1]. The most common tumors giving rise to secondary tumors of the ovaries (STOs) include breast, colorectal, endometrial, stomach, and appendix cancer, with the incidence varying considerably across different geographical regions of the globe [1]. Colorectal cancer (CRC) accounts for 12.5–41.2% of STOs, and it is the most common cause of STOs in Western countries [2,3,4,5,6,7,8]. It is generally accepted that tumors originating from the right versus left side of the colon/rectum have different embryogenic origins, molecular characteristics, histology, and prognosis [9]. However, little is known regarding the effect of the primary tumor sidedness on the clinicopathological characteristics of STOs. Despite a generally unfavorable prognosis of STOs, some patients may gain benefits from metastasectomy provided no residual tumor is achieved [10]. The identification of potential prognostic factors might help select the best candidates for surgery. We performed a retrospective study to evaluate the differences in the clinicopathological characteristics of STOs originating from right- and left-sided primary CRC and searched for potential prognostic markers. The secondary aim of our study was to evaluate the sensitivity of imaging methods in the diagnostics of STOs, with emphasis on their accuracy in determining the laterality of STOs.

## 2. Materials and Methods

### 2.1. Study Population

The histopathological registry of The Fingerland Department of Pathology, University Hospital Hradec Králové, was used to identify patients with tumors metastatic to the ovaries diagnosed between January 2000 and December 2019. A flow chart of the patient selection is shown in Supplementary Figure S1. Only patients with histologically confirmed STOs of colorectal origin (excluding cases with direct tumor spread to the ovaries) who were treated in our center were included in the study. A retrospective chart review was performed to obtain individual clinical and histopathological data.

Right- and left-sided primary tumors were defined as having their origin proximally or distally from the distant third of the transverse colon, respectively. The disease was characterized as synchronous if the time interval between primary tumor diagnosis and STO detection was less than six months and metachronous if the interval exceeded six months. Overall survival (OS) was calculated from the date of diagnosis of the STO to the time of death of any cause. Patients were followed until death or the end of follow-up (February 2020). Living patients were censored at their last follow-up visit. A preoperative radiologic assessment was performed with CT, MRI, PET/CT, or ultrasound, and the findings were compared with the histopathological reports. In the minority of cases where unilateral adnexectomy/ovariectomy was performed, information regarding the laterality of the STOs was derived from intraoperative findings supplemented by follow-up investigations (i.e., relapse in the preserved ovary documented <6 months after the surgery would be considered an initial bilateral STO).

The histopathological data included the STO laterality (unilateral vs. bilateral), gross morphology, histological type, tumor grade, presence of mucinous component, necrosis, and the classification of the Krukenberg tumor. Histopathological diagnosis was made according to the International Classification of Diseases for Oncology, Third Edition (ICD-O-3) [11]. Krukenberg tumors were identified using the diagnostic criteria of the World Health Organization (WHO) based on the pathological description by Serov and Scully [12]. The data used was anonymous, and the study was approved by the Ethics Committee of the University Hospital in Hradec Králové. The study was performed following relevant national and international guidelines and regulations.

### 2.2. Statistical Analysis

Descriptive statistics were used to summarize the patient and treatment characteristics. The clinicopathological variables were compared using Fisher’s exact test, the chi-square (χ^2^) test for trend, and a two-sample t-test when appropriate. Survival was analyzed using the Kaplan–Meier method and compared using the log-rank test. All these tests were two-sided, and *p* < 0.05 was considered statistically significant.

Univariable and multivariable Cox proportional hazard models were used to investigate associations between selected variables and survival. In the univariable analysis, we investigated the following variables: age (<50 years, ≥50 years), menopausal status, the topology of the primary tumor (colon vs. rectum), tumor sidedness, the chronology of metastatic disease, the presence of extraovarian metastases, peritoneal carcinomatosis, the presence of ascites, the laterality of STO, gross appearance, tumor grade, the presence of a mucinous component, signet ring cells, residual disease, systemic therapy, targeted therapy, and histopathological characteristics. All variables with *p* < 0.2 (Wald test) were included in the multivariable analysis. Some of these variables were correlated (age with menopausal status and residual disease with extraovarian metastases (Cramer’s V = 0.83 and 0.90, respectively)), and this, as well as the low number of events relative to the number of variables, could undermine the stability of a model based on ordinary least squares (OLS). With regard to this, we used elastic net penalized Cox proportional hazards regression with cross-validation. Penalization methods mitigate the impact of collinearity on the stability of the regression coefficient estimates by shrinking their size. The degree of shrinkage is controlled through a penalty parameter λ. The elastic net penalty is a linear combination of lasso and ridge regression penalties. Ridge regression shrinks the coefficients but not completely to zero; thus, it does not eliminate any variables. Meanwhile, the lasso may scale the coefficients completely to zero, which leads to the more influential variables being selected. However, if some predictors are highly correlated, the lasso will only keep one of them and entirely ignore the other. The elastic net penalization combines the advantages of ridge and lasso. The penalty parameter λ was evaluated through cross-validation. Suitable λ and linear combinations of lasso and ridge penalties were determined based on partial likelihood deviance.

The association between survival and the serum carcinoembryonic antigen (CEA) concentration at the time of STO diagnosis was investigated on a subgroup of 29 patients where the CEA values were available. With regard to the small size of this subgroup, only a univariable Cox regression analysis was performed.

Statistical analyses were performed using NCSS 10 Statistical Software (2015, NCSS, LLC, Kaysville, UT, USA) and R 4.0.4 (2021, R Foundation for Statistical Computing, Vienna, Austria) glmnet package 4.1-1 [13].

## 3. Results

### 3.1. Patient Characteristics

We identified 51 patients who fulfilled the preplanned inclusion criteria (see Figure S1). The median age of all the patients with CRC metastatic to the ovary was 58.6 years (range 35.1–84.2 years). The number of patients with synchronous and metachronous disease was similar (29 versus 22 patients). Fifty patients underwent surgery for STO, and concurrent resection of the primary tumor was performed in 21 cases (see Table S1). Systemic therapy was given to 38 (75%) patients, including those with macroscopic residual disease and those who relapsed after radical surgery. Detailed patient characteristics are shown in Table 1.

The serum concentrations of the tumor markers cancer antigen 125 (CA 125) and carcinoembryonic antigen (CEA) at the time of STO diagnosis were available in 67% and 57% of the patients, respectively (45% for both the tumor markers simultaneously). CA 125 and CEA were elevated in 50% (17/34) and 79% (23/29) of the cases, respectively. The median CA 125/CEA ratio was 0.71 (range 0.03–83.45), and this was lower than the cut-off value of 25 in 87.0% (20/23) of the cases.

### 3.2. Clinicopathological Characteristics of STOs and Primary Tumors

Of all the CRC patients investigated, there were 31 patients (61%) with left-sided and 20 patients (39%) with right-sided CRC. The most frequent primary site of CRC was the sigmoid colon (28%), and the predominant histological type was adenocarcinoma not otherwise specified (NOS, 78%). Further detailed primary CRCs are listed in Table S2.

At the time of STO diagnosis, the majority of patients had concurrent extraovarian metastatic spread (i.e., the presence of peritoneal carcinomatosis and/or distant metastases). When comparing right- and left-sided CRC, we could not find any significant differences in terms of extraovarian spread or the proportion of synchronous and metachronous disease. However, distant metastases (excluding peritoneal spread) were more common in the left-sided primary tumors (55% vs. 25%, *p* = 0.046). On the other hand, peritoneal carcinomatosis and ascites were more common in STOs arising from right-sided primary tumors. Bilateral involvement of the ovaries in the STOs was documented in 23 patients (45%) and was significantly more common in right-sided tumors (Table 2). In the case of unilateral STO, the metastasis was located in the left and right ovary in 39% and 61% of cases, respectively. We could not observe any association between the primary tumor sidedness and the STO laterality (i.e., right-sided primary tumors showed no propensity to form unilateral STOs in the right ovary and likewise in the left-sided tumors).

From a histopathological point of view, there was no difference in the size of ovaries originating from right- versus left-sided CRC. However, tumor affection of the ovarian surface was detected more frequently in STOs originating from right-sided primary tumors (58% vs. 25%, *p* = 0.034). A mucinous component was detected in 31% of the STOs and was significantly more common in tumors arising from the right colon (50% vs. 19%, *p* = 0.031). Necrosis was present in 55% and 30% of STOs arising from the left- and right-sided primary CRC, respectively, but this difference was not statistically significant (*p* = 0.095). The pathological STO characteristics are listed in Table 2.

### 3.3. Radiologic Assessment and Its Concordance with the Histopathological Report

Preoperative radiological assessment was available in 46 patients. In 11 of these patients (24%), the STO diagnosis preceded the diagnosis of the primary tumor. Importantly, in 21 patients (52%), the radiological findings were at first considered to indicate primary ovarian cancer. This was significantly more frequent when the ovarian mass was diagnosed synchronously (versus metachronously) with the primary tumor.

Interestingly, there was low concordance between the laterality diagnosed via imaging methods and the histopathological investigation and/or intraoperative findings. The imaging methods reported no visible adnexal mass in 13% of patients. It should be noted that in the vast majority of such cases (83%), the histopathological investigation revealed only micrometastatic disease. The presence of a pelvic mass (without any information regarding laterality) was reported in 26% of patients. Furthermore, in 15% of cases, the imaging methods showed unilateral ovarian involvement, which was proven to be bilateral according to the histopathological report and/or intraoperative findings. Overall, in 54% of cases, the preoperative radiological assessment was not able to correctly determine the laterality of ovarian involvement. More information regarding the imaging methods and their comparison with the histopathological findings can be found in Table S3.

We also assessed the value of the intraoperative findings in the detection of STOs. The STO laterality was correctly determined in 80% of cases based on the intraoperative findings. Bilateral STOs were misdiagnosed as unilateral in 10% of cases, and, in a single case of bilateral STO, normal gross appearance was documented. Interestingly, 8% of unilateral STOs were intraoperatively assessed as bilateral (histopathological evaluation of both ovaries was performed in all of these cases).

### 3.4. Survival and Prognostic Factors

**Figure 1:**
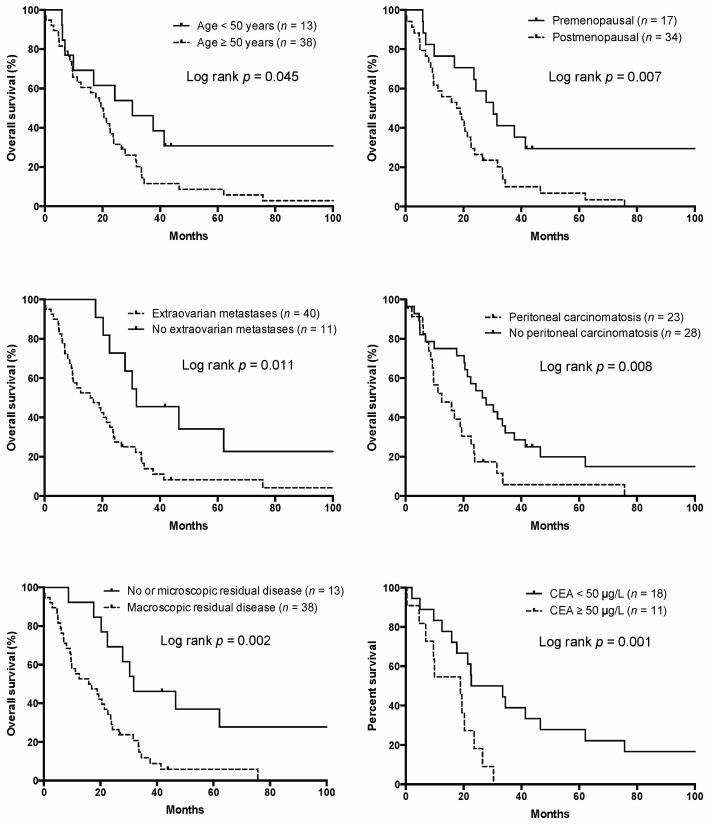
Overall survival according to the risk factors.

At the time of analysis, six patients were alive, with a median follow-up time of 81.1 months (range 26.9–183.5 months), while 45 patients had died. The median OS was 20.5 months (range 0.1–183.5). In a univariable Cox regression analysis, age, menopausal status, topology of the primary tumor, the presence of extraovarian metastases, peritoneal carcinomatosis, CEA serum concentration (<50 µg/L vs. ≥50 µg/L), signet-ring cell morphology, and residual disease after cytoreductive surgery, administration of systemic therapy, and the use of targeted therapy were identified as potential prognostic factors for OS (Table 3 and Figure 1). Univariable analysis on a subgroup of 29 patients identified a CEA serum concentration ≥50 µg/L as a prognostic factor for OS (*p* = 0.001, HR = 3.47, 95% CI 1.16–10.32).

In a multivariable analysis, the presence of macroscopic residual disease after cytoreductive surgery was identified as the most influential adverse risk factor for OS, followed by menopausal status and presence of extraovarian metastases, whereas the administration of systemic therapy and targeted therapy were identified as protective factors (Table 3).

## 4. Discussion

Ovarian metastases from solid tumors are far from rare, representing 10–20% of all ovarian tumors. In Western countries, metastases to the ovaries are commonly derived from colorectal and breast cancer [1,6]. In our study, colorectal cancer was the primary tumor site in 40.1% of STOs, which approaches the high-end threshold reported by other authors (12.5–41.2%) [2,3,4,5,6]. This can probably be attributed to a high incidence of CRC in the Czech Republic [14], as the rate of different primary tumors responsible for STOs reflects their incidence in the general population [5]. The median age at STO diagnosis in our series was lower than that reported in the general female population with metastatic colorectal cancer (59 vs. 73 years) [15]. Correspondingly, the proportion of premenopausal women with STOs was higher than in the general metastatic CRC (mCRC) population [16], which is consistent with previous reports [17,18]. Premenopausal women with CRC are more likely to develop ovarian metastases than their postmenopausal counterparts [19]. This can be explained by the fact that functional ovaries have a rich ovarian blood supply, which might facilitate hematogenous spread [20].

Despite a relatively high incidence of STOs, their diagnosis is rather challenging, because up to 45% of ovarian metastases from CRC are initially diagnosed as primary ovarian cancer [7,21,22]. Moreover, STOs of colorectal origin have been reported to be the most common tumors mimicking primary ovarian tumors (32–36% of STOs presenting as primary ovarian cancer) [3,8]. Consistently, 47% of the patients in our study were initially diagnosed as having primary ovarian tumors. The ratio was even higher in patients presenting with ovarian metastases (67%). The diagnosis of ovarian metastases precedes the detection of the primary tumor in up to 38–42% of cases, which might prompt the misdiagnosis of STOs as primary ovarian tumors [23,24]. We observed a similar circumstance in our study (22%). Moreover, 12% of the patients with STOs had no visible adnexal mass upon imaging in our series. In comparison with the histopathological analysis, the imaging methods used in these patients allowed for correct STO laterality assessment in only 46% of cases. Further, 15% of bilateral STOs were misdiagnosed as unilateral by these imaging methods, thus highlighting their inadequacy in ruling out contralateral ovarian metastases. In accordance with our results, high rates of radiologically occult STOs originating from CRC have been reported by other authors [25,26,27]. Huang et al. reported that almost one-quarter of patients with grossly normal ovaries had microscopic metastatic disease [25]. CT is considered a standard imaging method before surgery for both primary ovarian cancer and metastatic disease originating from different primary tumors. Accordingly, CT was the most commonly used imaging method in our study. Although this modality represents an excellent tool in the diagnosis of advanced disease, its ability to detect very small lesions is limited [28]. Therefore, the possibility of micrometastatic disease in ovaries and the limited sensitivity of CT in detecting small lesions could serve as an explanation for its modest performance in the detection of STOs. On the other hand, voluminous STOs are commonly described as a pelvic mass, and the determination of laterality may not be possible using imaging methods. Intraoperative findings can provide additional information in such cases. However, neither intraoperative inspection by the surgeon is able to detect microscopic STOs in grossly normal ovaries.

Based on our findings and the available literature, a bilateral adnexectomy should be encouraged in all postmenopausal patients with unilateral STOs diagnosed by imaging methods [29,30]. In premenopausal patients, the possibility of unilateral adnexectomy should be discussed with the patient on an individual basis, especially if fertility preservation is desired. The patient, however, needs to be fully informed of the risk of occult micrometastases in the contralateral ovary and the possibility of developing metachronous metastases in the preserved ovary. Intraoperative assessment of the preserved ovary with the possible use of frozen section biopsy should be performed in such cases to rule out possible tumor involvement [29].

One of the patients in our study underwent unilateral adnexectomy for a STO and consecutively developed a metachronous recurrence in her contralateral ovary with a relapse-free survival of 8.1 months. Such cases have been previously reported in the literature and provide yet another reason for performing a bilateral adnexectomy—even in cases of unilateral STOs—to prevent the patient from undergoing additional laparotomy and tumor resection for metachronous recurrence [31].

Taking into account the limited ability of imaging methods to distinguish primary ovarian tumors from STOs, additional investigations have been evaluated in this regard. One of such investigations was a preoperative serum CA 125/CEA ratio with a cut-off value of 25 [32]. In our study, 87% of the patients with known CA 125 and CEA levels had a CA 125/CEA ratio lower than 25, thus supporting the use of this ratio as an auxiliary diagnostic tool.

It is now clear that the right and left colon have different embryonic origins, thus explaining why the tumors originating from them display diverging mutations and metastatic patterns [9]. While left-sided colorectal cancers usually give rise to liver and lung metastases, right-sided primary tumors have a high propensity to develop peritoneal metastases [33]. Although hematogenous spread is considered the predominant pathway of metastatic dissemination into the ovaries in colorectal cancer [3,34], it seems probable that other metastatic routes (including lymphogenous and peritoneal spread) can also take part. Furthermore, different metastatic pathways may combine, especially in the case of more advanced disease [34]. In our study, we found that peritoneal carcinomatosis, bilateral ovarian metastases, and tumor involvement of the ovarian surface were significantly more frequent in right-sided primary tumors. Conversely, left-sided primary tumors formed distant metastases (excluding peritoneal spread) and unilateral ovarian metastases at a higher frequency, although there has been no statistical difference concerning left and right ovarian involvement. These findings are consistent with those observed by other authors [35,36,37,38]. Interestingly, peritoneal carcinomatosis was less frequent and the ovarian surface was more frequently devoid of tumor cells in the left-sided primary tumors. Therefore, it could be hypothesized that right- and left-sided primary tumors might preferably develop STOs through different metastatic pathways. While hematogenous and/or lymphogenous spread seems to be responsible for the majority of STOs of left-sided origin, peritoneal dissemination might be the predominant metastatic pathway in right-sided primary tumors. Notably, we could not observe any statistically meaningful difference in the proportion of T4 tumors between right-sided and left-sided primary CRC (*p* = 0.764). Therefore, the penetration through the visceral peritoneum itself probably cannot explain the propensity of right-sided colon cancer to develop peritoneal metastases. Other factors known to be associated with peritoneal spread, including mucinous histology, the presence of *BRAF* activating mutations, and poor differentiation are more frequently found in right-sided tumors [33,39,40,41]. The propensity of mucinous adenocarcinomas to form peritoneal metastases is well-documented and is explained by the production of mucus under pressure, which enables tumor cells to reach the peritoneal cavity and further support their spread in the form of gelatinous ascites [39]. The coincidence of peritoneal and ovarian metastases in right-sided tumors observed in our study is consistent with the results of a large population-based study [15]. This can be explained by anatomic factors and the fact that the peritoneum and ovarian stroma share the same embryogenic origin and molecular characteristics [42].

The survival of patients with STOs is generally poor, as they principally represent an advanced disease stage [4]. However, tumors of the colon have been repeatedly identified as a subgroup with better survival when compared to other primary gastrointestinal tract cancer [23,43,44,45]. The median OS in our study was 20.5 months, which is consistent with previously reported data [45,46,47]. Although the number of patients eligible for metastasectomy is limited, selected patients may derive survival benefits and achieve long-term remissions. In this regard, optimal cytoreduction surgery (as performed in primary ovarian tumors) results in a better prognosis even in patients with extraovarian CRC spread [48]. Accordingly, every effort should be directed toward achieving complete cytoreduction and preoperative evaluation by a tumor board should be encouraged to select the most appropriate candidates for surgery. Interestingly, patients with CRC-derived ovarian metastases have been reported to show worse responses to systemic therapies compared to those with extraovarian metastases [29,48,49], which puts forth an enhanced emphasis on the practice of metastasectomy whenever feasible.

As expected, the prognosis of younger and/or premenopausal women was significantly better compared to their older and/or postmenopausal counterparts. This finding is not limited to patients with ovarian metastases but was reported for the female population diagnosed with mCRC in general [16]. Although younger age and premenopausal status overlap, it seems that the menopausal status itself bears the main prognostic effect. In support of this hypothesis, young women aged <45 years have a significantly better prognosis than men of the same age, but this benefit does not extend to older women as they become postmenopausal [16]. It is probable that the higher estrogen level in premenopausal women has an anti-tumor effect through ERβ-mediated pro-apoptotic signaling, the inhibition of inflammatory signals, and the modulation of the tumor microenvironment [50].

Normal CEA levels are a favorable prognostic factor for OS [48]. In our study, only 21% of the 29 patients with available CEA values had normal CEA levels at the time of STO diagnosis; meanwhile, 62% had CEA ≥ 50 µg/L, and this was found to be an adverse prognostic factor for OS upon univariable analysis. A cut-off value of 50 µg/L (as suggested by other authors [51,52,53]) was used instead of normal vs. elevated (i.e., >5 µg/L) serum CEA concentrations to provide a more even patient distribution and reflect the fact that mild elevations may not be tumor-related. On this basis, we propose this cut-off value instead of normal vs. abnormal CEA serum concentrations and suggest preoperative CEA levels to be considered in the selection of resection surgery candidates. Even if this factor seems promising, it was not included in the multivariable analysis due to a lower number of patients with available preoperative serum CEA concentrations (*n* = 29).

Patients who were treated with systemic chemotherapy and/or targeted therapy had better OS than untreated patients in our study. This unsurprising finding can be attributed to both the effect of therapy itself but also to the fact that systemic therapy—especially targeted therapy—is reserved for patients in a better condition.

This study had certain limitations, mainly its limited sample size (which could hinder the identification of additional prognostic factors) and retrospective nature. Moreover, the extended period of the study complicates the survival analysis, mostly due to the introduction of new targeted therapy agents during its course.

## 5. Conclusions

The potential presence of a secondary ovarian tumor-derived from colorectal cancer should always be considered in patients with a pelvic mass. Although the role of cytoreductive surgery is not clear, it should always be considered in patients with a disease confined to the ovaries and where complete resection is feasible. Further, a resection of both ovaries should be performed in all postmenopausal women even in cases of a unilateral mass when diagnosed through imaging methods, because occult micrometastases in the contralateral ovary are frequently overlooked by these methods. This observation holds especially true in the case of right-sided primary tumors, in which the bilateral metastatic involvement is more frequent. If unilateral adnexectomy is considered in premenopausal women to preserve fertility, thorough information regarding the potential risks should be provided. The results of our study suggest that, while hematogenous and/or lymphogenous spread to the ovaries seems to be the preferred metastatic pathway from left-sided primary tumors, peritoneal spread might be the more common pathway of right-sided primary tumors. However, more extensive research is clearly needed to support this hypothesis.

## Figures and Tables

**Table 1:** Demographical and clinical characteristics according to the primary tumor site.

Characteristics	Right-Sided (*n* = 20)	Left-Sided (*n* = 31)	Total (*n* = 51)	*p*-Value
Age (mean ± SD)	58.8 ± 11.2	59.2 ± 13.9	59.0 ± 12.7	0.915 *
Menopausal status				
Premenopausal	5	12	17 (33.3%)	0.373 ^†^
Postmenopausal	15	19	34 (66.7%)	
Chronology				
Synchronous	13	16	29 (56.9%)	0.397 ^†^
Metachronous	7	15	22 (43.1%)	
Supposed primary ovarian cancer				
Yes	12	12	24 (47.1%)	0.161 ^†^
No	8	19	27 (52.9%)	
Extraovarian metastases ^1^				
Yes	15	25	40 (78.4%)	0.733 ^†^
No	5	6	11 (21.6%)	
Distant metastases ^2^				
Yes	5	17	22 (43.1%)	**0.046** ^†^
No	15	14	29 (56.9%)	
Ascites				
Absent or <500 mL	8	21	29 (56.9%)	0.071 ^‡^
500–1000 mL	7	6	13 (25.5%)	
>1000 mL	5	4	9 (17.6%)	
Peritoneal carcinomatosis				
Present	14	9	23 (45.1%)	**0.009** ^†^
Absent	6	22	28 (54.9)	
Residual disease after surgery				
None (R0) or microscopic (R1)	6	7	13 (25.5%)	0.743 ^†^
Macroscopic (R2)	14	24	38 (74.5%)	
Systemic therapy ^3^				
Yes	20	22	42 (82.4%)	**0.008** ^†^
No	0	9	9 (17.6%)	
Targeted therapy				
Yes	13	14	26 (51.0%)	0.251 ^†^
No	7	17	25 (49.0%)	
Overall survival (median, range) ^4^	21.0(6.1–183.5)	20.5(0.1–118.4)	20.5(0.1–183.5)	0.455 ^§^

Significant *p*-values in bold, STO–secondary tumor of the ovary, SD–standard deviation. ^1^ Presence of peritoneal carcinomatosis and/or distant metastases, ^2^ Excluding peritoneal spread, ^3^ Systemic therapy–adjuvant and/or palliative chemotherapy/targeted therapy, ^4^ Overall survival from the date of diagnosis of STO until death of any cause, * Two-sample *t*-test, ^†^ Fisher’s exact test, ^‡^ χ^2^ test for trend, ^§^ Kaplan–Meier method with a log-rank test.

**Table 2:** Pathological characteristics of STO.

Characteristics	Right-Sided (*n* = 20)	Left-Sided (*n* = 31)	Total (*n* = 51)	*p*-Value
Laterality				
Unilateral	6	22	28 (54.9%)	**0.009** *
Bilateral	14	9	23 (45.1%)	
Ovary–diameter				
<5 cm	3	6	9 (17.6%)	0.593 ^‡^
5–10 cm	7	10	17 (33.3%)	
>10 cm	8	10	18 (35.3%)	
Not reported	2	5	7 (13.7%)	
Gross appearance				
Solid	8	8	16 (31.4%)	0.455 ^†^
Cystic	4	9	13 (25.5%)	
Mixed	4	10	14 (27.5%)	
Not reported	4	4	8 (15.7%)	
Ovarian surface ^1^				
Smooth, free of tumor	8	21	29 (61.7)	**0.034** *
Affected with tumor	11	7	18 (38.3)	
Histology				
Adenocarcinoma NOS	13	26	39 (76.5%)	0.327 ^†^
Mucinous adenocarcinoma	6	4	10 (19.6%)	
Poorly cohesive/Signet-ring cell adenocarcinoma	1	1	2 (3.9%)	
Tumor grade				
1	3	0	3 (5.9%)	0.109 ^‡^
2	12	20	32 (62.7%)	
3	5	11	16 (31.4%)	
Mucinous component				
Present	10	6	16 (31.4%)	**0.031** *
Absent	10	25	35 (68.6%)	
Necrosis				
Present	6	17	23 (45.1%)	0.095 *
Absent	14	14	28 (54.9%)	

STO–secondary tumor of the ovary, NOS–not otherwise specified, significant *p*-values in bold. ^1^ Information regarding tumor affection of the ovarian surface was available in 47 patients, * Fisher’s exact test, ^†^ Extended Fisher’s exact test, ^‡^ χ^2^ test for trend.

**Table 3:** Prognostic factors for overall survival.

Prognostic Variables	Univariable Analysis		Multivariable Analysis	
	HR (95% CI)	*p*-Value	Regression Coefficient	HR
Age				
<50 years (*n* = 13)	Ref.			
≥50 years (*n* = 38)	2.10 (1.00–4.41)	0.050	-	-
Menopausal status				
Premenopausal (*n* = 17)	Ref.			
Postmenopausal (*n* = 34)	2.45 (1.25–4.81)	0.009	0.141	1.15
Colon vs. rectum				
Colon (*n* = 44)	Ref.			
Rectum (*n* = 7)	1.79 (0.79–4.07)	0.163	-	-
Tumor sidedness				
Right-sided (*n* = 20)	Ref.			
Left-sided (*n* = 31)	1.26 (0.68–2.33)	0.456		
Chronology				
Synchronous (*n* = 29)	Ref.			
Metachronous (*n* = 22)	1.18 (0.66–2.13)	0.572		
Extraovarian metastases				
No (*n* = 11)	Ref.			
Yes (*n* = 40)	2.66 (1.22–5.78)	0.014	0.011	1.01
Peritoneal carcinomatosis				
No (*n* = 28)	Ref.			
Yes (*n* = 23)	2.24 (1.21–4.12)	0.010	-	-
Ascites				
No (*n* = 21)	Ref.			
Yes (*n* = 30)	1.14 (0.63–2.07)	0.667		
Laterality of STO				
Unilateral (*n* = 27)	Ref.			
Bilateral (*n* = 24)	0.85 (0.47–1.53)	0.580		
Histological type				
Adenocarcinoma (*n* = 39)	Ref.			
Mucinous adenocarcinoma (*n* = 10)	1.03 (0.49–2.15)	0.937	-	-
Signet ring cell carcinoma (*n* = 2)	5.75 (1.29–25.61)	0.022	-	-
Tumor grade				
1 (*n* = 3)	Ref.			
2 (*n* = 32)	1.50 (0.35–6.38)	0.582		
3 (*n* = 16)	2.08 (0.47–9.29)	0.337		
Mucinous component				
No (*n* = 35)	Ref.			
Yes (*n* = 16)	1.35 (0.72–2.52)	0.350		
Signet ring cells				
No (*n* = 42)	Ref.			
Yes (*n* = 9)	1.87 (0.89–3.96)	0.100	-	-
Residual disease				
No or microscopic residual disease (*n* = 13)	Ref.			
Macroscopic residual disease (*n* = 38)	3.11 (1.45–6.66)	0.004	0.288	1.33
Systemic therapy ^1^				
Omitted (*n* = 9)	Ref.			
Given (*n* = 42)	0.24 (0.11–0.50)	<0.001	−0.135	0.87
Targeted therapy				
Omitted (*n* = 25)	Ref.			
Given (*n* = 26)	0.48 (0.26–0.87)	0.015	−0.117	0.89
CEA serum concentration ^2^				
<50 µg/L (*n* = 18)	Ref.			
≥50 µg/L (*n* = 11)	3.16 (1.29–7.76)	0.012		

STO–secondary tumor of the ovary, HR–Hazard Ratio, CI–confidence interval, ^1^ Systemic therapy–adjuvant and/or palliative chemotherapy (with or without targeted therapy), ^2^ The analysis was performed in 29 patients with known carcinoembryonic antigen (CEA) serum concentration.

## Data Availability

The data supporting the findings of this study is available on request from the corresponding author, J.K.

## Supplementary Materials

The following are available online at https://www.mdpi.com/article/10.3390/curroncol28040255/s1, Table S1: Surgery for STO (*n* = 51), Table S2: Primary tumor characteristics (*n* = 51), Table S3: Imaging methods and their comparison with histopathological findings, Figure S1: Flow chart of exclusion process.
