# Supplementary material for: Clinicopathological Characteristics and Prognostic Factors in Ovarian Metastases from Right- and Left-Sided Colorectal Cancer
Source: Curr Oncol. 2021 Aug 3;28(4):2914–27. doi: 10.3390/curroncol28040255 (PMC8395391; doi:10.3390/curroncol28040255)
Supplement: Supplementary file 1 [file curroncol-28-00255-s001.zip › curroncol-1307372-supplementary.pdf]

Current Oncology

MDPI

Table S1. Surgery for STO (*n* = 51).

Type of procedure	Concurrent resection of the primary tumor	Number of patients (*n* = 51)
Biopsy	0	1 (2.0%)
Unilateral adnexectomy	6	10 (19.6%)
Bilateral adnexectomy	4	10 (19.6%)
Bilateral adnexectomy, hysterectomy	7	16 (31.4%)
Bilateral adnexectomy, hysterectomy, omentectomy	4	7 (13.7%)
Bilateral adnexectomy, hysterectomy, omentectomy, appendectomy	0	5 (9.8%)
Bilateral adnexectomy, hysterectomy, omentectomy, appendectomy, lymphadenectomy	0	1 (2.0%)
Palliative surgery (i.e. colonostomy, ileostomy, etc.)	0	1 (2.0%)

STO – secondary tumor of the ovary.

Table S2. Primary tumor characteristics (*n* = 51).

Characteristics	Right-sided (*n* = 20)	Left-sided (*n* = 31)	Total (*n* = 51)
Initial disease stage			
I	0	1	1 (2.0%)
II	1	5	6 (11.8%)
III	3	4	7 (13.7%)
IV	16	21	37 (72.5%)
Primary tumor stage			
≤ T3	9	16	25 (49.0%)
T4	9	12	21 (41.2%)
Unknown	2	3	5 (9.8%)
Regional lymph nodes			
Positive	9	21	30 (58.8%)
Negative	11	10	21 (41.2%)
Histology			
Adenocarcinoma NOS	7	28	35 (68.6%)
Mucinous adenocarcinoma	12	1	13 (25.5%)
Poorly cohesive/signet-ring cell	0	1	1 (2.0%)
Unknown	1	1	2 (3.9%)
Tumor grade			
1	2	0	2 (4.1%)
2	12	18	30 (61.2%)
3	5	12	17 (34.7%)
Unknown	1	1	2 (3.9%)
Mucinous component			
Present	12	6	18 (36.7%)
Absent	7	25	31 (63.3%)
Unknown	1	0	1 (2.0%)
Primary tumor site (ICD code)			
Colon total (C18)			44 (86.3%)
Coecum (C18.0)			7 (13.7%)
Appendix (C18.1)			5 (9.8%)

Ascending colon (C18.2)	6 (11.8%)
Hepatic flexure (C18.3)	2 (3.9%)
Transverse colon (C18.4)	0 (0%)
Splenic flexure (C18.5)	2 (3.9%)
Descending colon (C18.6)	0 (0%)
Sigmoid colon (C18.7)	14 (27.5%)
Rectosigmoid junction (C19)	8 (15.7%)
Rectum (C20)	7 (13.7%)

ICD—International Classification of Diseases, NOS – not otherwise specified.

Table S3. Imaging methods and their comparison with histopathological findings.

Imaging methods available	46 (90.2%)
Imaging methods used [1]	
CT	37 (80.4%)
MRI	6 (13.0%)
US	5 (10.9%)
PET/CT	3 (6.5%)
Suggestion of imaging methods	
Primary ovarian cancer	21 (45.7%)
STO	25 (54.3%)
Findings on imaging methods	
No visible adnexal mass	6 (13.0%)
Pelvic mass [2]	12 (26.1%)
Unilateral adnexal mass	19 (41.3%)
Bilateral adnexal mass	9 (19.6%)
Accuracy of imaging methods	
Correctly evaluated	21 (45.7%)
Bilateral misdiagnosed as unilateral	7 (15.2%)
Information on laterality missing [2]	12 (26.1%)
No visible adnexal mass	6 (13.0%)

Imaging methods were available in 46/51 (90.2%) patients, [1] multiple imaging methods in one patients might have been used, [2] reported as pelvic mass with no information on laterality.

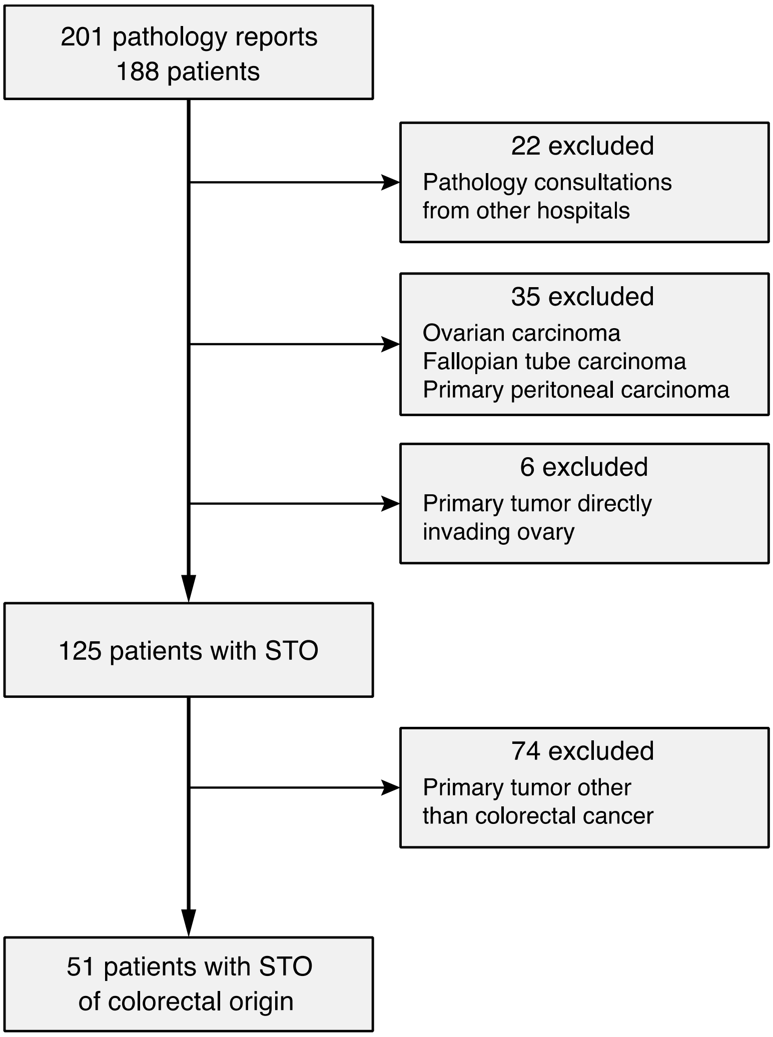

Figure S1. Flow chart of exclusion process.
